# Quantitation of Tissue Amyloid via Fluorescence Spectroscopy Using Controlled Concentrations of Thioflavin-S

**DOI:** 10.3390/molecules28114483

**Published:** 2023-06-01

**Authors:** Tatiana P. MacKeigan, Megan L. Morgan, Peter K. Stys

**Affiliations:** Department of Clinical Neurosciences, Hotchkiss Brain Institute, Cumming School of Medicine, University of Calgary, 3330 Hospital Drive NW, Calgary, AB T2N 4N1, Canada; tatiana.mackeigan@ucalgary.ca (T.P.M.); megan.morgan1@ucalgary.ca (M.L.M.)

**Keywords:** Thioflavin-S, fluorescence spectroscopy, amyloid, Alzheimer’s disease

## Abstract

Amyloids are misfolded proteins that aggregate into fibrillar structures, the accumulation of which is associated with the pathogenesis of many neurodegenerative diseases, such as Alzheimer’s disease (AD). Early, sensitive detection of these misfolded aggregates is of great interest to the field, as amyloid deposition begins well before the presentation of clinical symptoms. Thioflavin-S (ThS) is a fluorescent probe commonly used to detect amyloid pathology. Protocols for ThS staining vary, but they often use high staining concentrations followed by differentiation, which causes varying levels of non-specific staining and potentially leaves more subtle amyloid deposition unidentified. In this study, we developed an optimized ThS staining protocol for the sensitive detection of β-amyloids in the widely used 5xFAD Alzheimer’s mouse model. Controlled dye concentrations together with fluorescence spectroscopy and advanced analytical methods enabled not only the visualization of plaque pathology, but also the detection of subtle and widespread protein misfolding throughout the 5xFAD white matter and greater parenchyma. Together, these findings demonstrate the efficacy of a controlled ThS staining protocol and highlight the potential use of ThS for the detection of protein misfolding that precedes clinical manifestation of disease.

## 1. Introduction

Protein misfolding and assembly into fibrillar structures are a hallmark of multiple central nervous system (CNS) disorders that are pervasive among the aging population [1]. Amyloids are misfolded proteins that aggregate and adopt a characteristic cross-β structure [2,3]. These structures are formed when repetitive arrays of β-sheets are oriented perpendicular to the fibril axis [4,5]. Amyloid accumulation in the CNS is associated with the pathogenesis of many neurodegenerative diseases, including Alzheimer’s disease (AD) and Parkinson’s disease [6,7]. Given that amyloid deposition begins well before the onset of clinical symptoms, the early and sensitive detection of these protein assemblies is of great interest [8,9].

Fluorescent amyloid probes are histological tools commonly used to visualize senile plaques and neurofibrillary tangles associated with Alzheimer’s disease. A wide array of these probes is available, including the thioflavins Thioflavin-S (ThS) and Thioflavin-T (ThT), Congo red, molecules in the oligothiophene family, and others [10,11,12,13,14]. Although these dyes lack specificity for particular protein amino acid sequences, they preferentially bind to misfolded protein aggregates by embedding themselves between stacks of highly ordered β-sheet structures [15,16]. Once bound, these probes become sterically hindered, and this increases their quantum yield and induces unique fluorescent emission patterns [17,18]. Thioflavins are of particular interest due to their characteristic shift of emission spectrum when binding to β-sheet motifs [19,20].

Thioflavins are synthesized from the methylation of dehydrothiotoluidine [21]. Specifically, ThS is produced by methylation via sulfonic acid. This reaction produces chemical grade ThS, which is a mixture of compounds that remain incompletely characterized. Amyloid deposits are identified by their green–yellow fluorescence, and a peak emission at 455 nm is observable for the most commonly used staining protocol [21]. Protocols for ThS staining vary, but they commonly employ a high concentration of dye for a short incubation, followed by differentiation using ethanol or other solvents [22,23,24,25], and this may result in varying levels of non-specific staining and inconsistent reproducibility. Differentiation can also cause the removal of ThS from potentially informative low-affinity binding sites, and this may lead to the under-detection of subtle yet relevant amyloid accumulation.

A number of fluorescent probes have been intentionally designed to change their emission intensity or spectrum based on pH, polarity, and their immediate environment [26]. Interestingly, while the spectral shift of ThT upon binding to amyloid fibrils is well characterized [27,28,29,30], less is known about the capacity of ThS to change its emission spectrum in the presence of amyloid aggregates.

In this study, we investigated the sensitivity with which ThS can detect β-amyloids in the 5xFAD Alzheimer’s mouse model. This transgenic mouse was created by inserting five genes for the accelerated deposition of β-amyloid in the brain and spontaneously develops robust amyloid plaque pathology in a predictable, age-dependent manner [31,32]. We developed an optimized ThS staining protocol with controlled dye concentrations that enabled a more effective visualization of amyloid pathology in the brain of young and old 5xFAD mice than traditional staining protocols using this dye. Additionally, spectral imaging of the ThS-stained tissues yielded more biologically relevant information than traditional fluorescence imaging, and together with advanced analytical methods, enabled the detection of subtle and widespread protein misfolding in the 5xFAD brain.

## 2. Results

### 2.1. Traditional ThS Staining Protocols Revealed Conventional Features in 5xFAD Brain 

**Figure 1:**
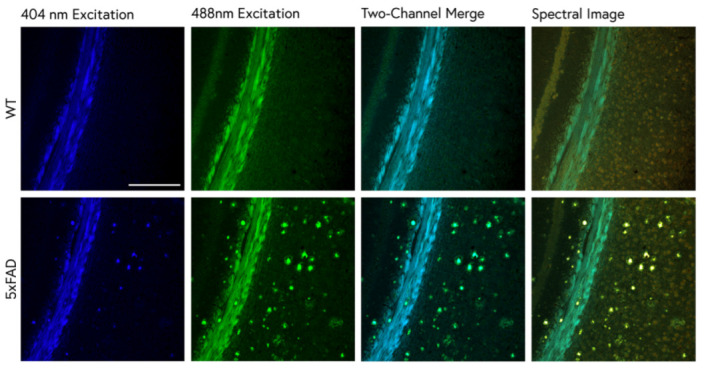
Brain sections from 9-month-old WT and 5xFAD mice stained with 1% ThS. The fluorescence emission of ThS provides more information when imaged in spectral mode than dual-channel imaging. Dual-channel images were collected with the DAPI and FITC emission filters and compared with spectral true-color images (**right**). Bright staining of the white matter (corpus callosum) prevented the identification of plaque pathology when imaged with one channel. Spectral imaging showed differential spectra of the white matter and plaque staining and also revealed faint, reddish cell bodies throughout the cortex. Scale bar: 200 μm.

**Figure 2:**
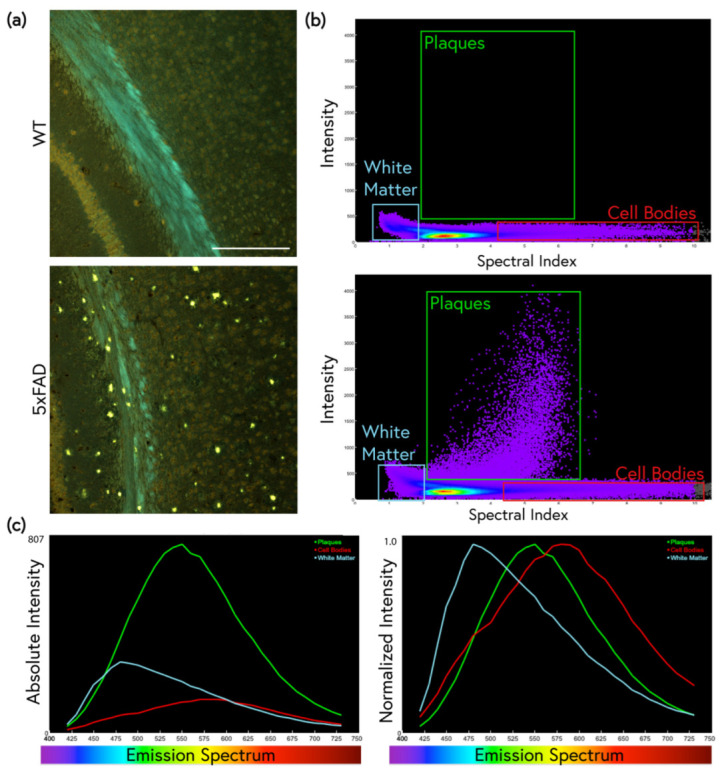
Spectral imaging differentiates between various tissue elements stained with 1% ThS. (**a**) Representative images of WT and 5xFAD brains. Scale bar: 200 μm. (**b**) Identification of relevant kernel clusters of aggregated WT and 5xFAD spectral images using spectral scatter analysis. The plaque pathology, blue-shifted white matter, and red-shifted cell bodies can be identified by their unique intensity and spectral characteristics. (**c**) These pixel populations are plotted on spectral graphs comparing absolute intensity (**left**) and normalized intensity (**right**). In terms of intensity, the plaques are the brightest feature, although the white matter is brighter than the background. Normalized graphs show that each element has a unique emission peak.

WT and 5xFAD tissue sections stained with ThS were compared using dual-channel and spectral confocal microscopy. When stained with a commonly employed 1% ThS protocol [23], bright amyloid plaque cores were visible throughout the 5xFAD cortex, as was expected. The white matter of both the WT and 5xFAD mice, however, was also brightly stained (Figure 1). When the same brain region was imaged using 32-channel spectral detection, several gray and white matter elements displayed unique spectral emission patterns and could be differentiated by both intensity and spectral shape. Isolation of specific kernel populations using spectral scatter analysis enabled the quantification of ThS-stained plaque pathology based on intensity and emission spectrum (Figure 2a,b). When different kernel populations were isolated and the average was spectrum plotted, white matter, plaques, and cell bodies were found to exhibit unique brightness and spectral patterns (Figure 2c). 

### 2.2. An Improved ThS Staining Protocol for Subtle Amyloid Detection

**Figure 3:**
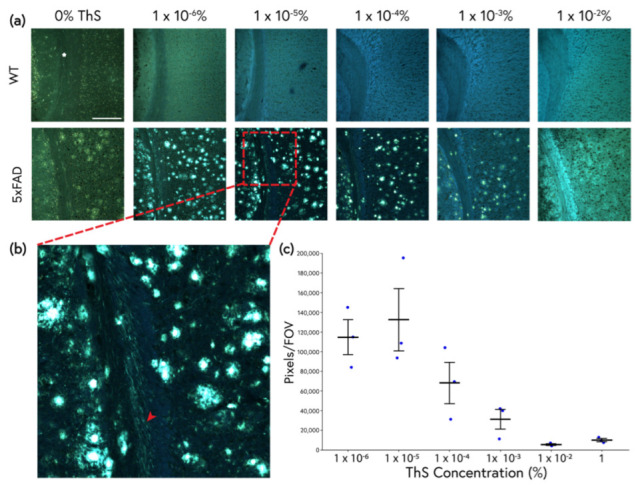
(**a**) Brain sections from 9-month-old WT and 5xFAD mice were stained with a broad range of ThS concentrations. Spectral images were not intensity-matched to allow better comparison of structural features across concentrations. Non-specific staining of the white matter (corpus callosum) was greatly reduced at lower concentrations. Plaque halos could be easily visualized at the lowest concentrations, and, interestingly, diffuse, thread-like pathology was present throughout the samples, particularly in the white matter. These features became more prominent as the concentration decreased. Scale bar: 200 μm. (**b**) Inset showing numerous thread-like deposits in the corpus callosum (arrow) and cortex that could not be observed at higher ThS concentrations. (**c**) Total area of bright ThS^+^ amyloid pathology as a function of ThS staining concentration. Quantification was carried out by selecting high-intensity pixels (all background and cell bodies omitted) identified on a spectral scatter plot and comparing the total number per FOV. Each dot on the graph represents a different 5xFAD mouse. The optimal ThS concentration for distinguishing amyloid deposits from background was 1 × 10^−5^%. Error bars represent SEM.

Mouse brains were stained using controlled concentrations of ThS by incubating sections in much lower ThS concentrations than are used in traditional protocols, but for a longer duration (overnight) in high volumes of staining solution. Importantly, all differentiation steps were deliberately omitted to avoid the removal of dye from potentially informative binding sites. Using this protocol, ThS was found to be sensitive across a wide concentration range for the detection of β-amyloid aggregates in the 5xFAD mice (Figure 3a). The quantity of visible amyloid pathology and the spectral character of the tissue depended heavily on the concentration used. Amyloid plaque cores were identifiable at nearly all ThS concentrations, wherein bright plaques contained red-shifted emission spectra in contrast with the bluer background parenchyma in the 5xFAD tissue. Plaques could be observed even at 0% ThS (see column 1 in Figure 3a), possibly because of intrinsic autofluorescence [33]. Importantly, ThS applied at optimized concentrations revealed far more intricate plaque pathology.

At 1 × 10^−2^% ThS (already 100 × below the traditional concentration of 1%), plaques were not readily identified due to the high background staining of the neuropil (hence the need to differentiate with ethanol when staining tissues at 1%). As the concentration of ThS was reduced, plaque cores and more subtle deposits (“halos”) extending well beyond the cores seen at 1% (Figure 1 and Figure 2) could be distinguished in the cortex. Linear thread-like pathology in the white matter (“white matter threads”) running parallel to axons became visible starting at 1 × 10^−4^% ThS and continuing to the lowest concentration tested (1 × 10^−6^% ThS, Figure 3b). At the lower concentrations, there was far less non-specific staining of the white matter compared with the 1% ThS protocol described above; at this high concentration, white matter exhibited such strong non-specific labeling that no white matter threads were visible (compare Figure 3a,b to Figure 2). Using spectral scatter analysis, the total ThS^+^ area of pathology was quantified by identifying relevant pixels using both spectrum and intensity. At concentrations of 1 × 10^−3^% to 1 × 10^−6^%, ThS revealed significantly more amyloid pathology than the conventional 1% ThS protocol due to the increased discriminability of the white matter threads and plaque halos. A concentration of 1 × 10^−5^% ThS was found to reveal the most pathology over background (Figure 3c) and was therefore selected for subsequent experiments. 

**Figure 4:**
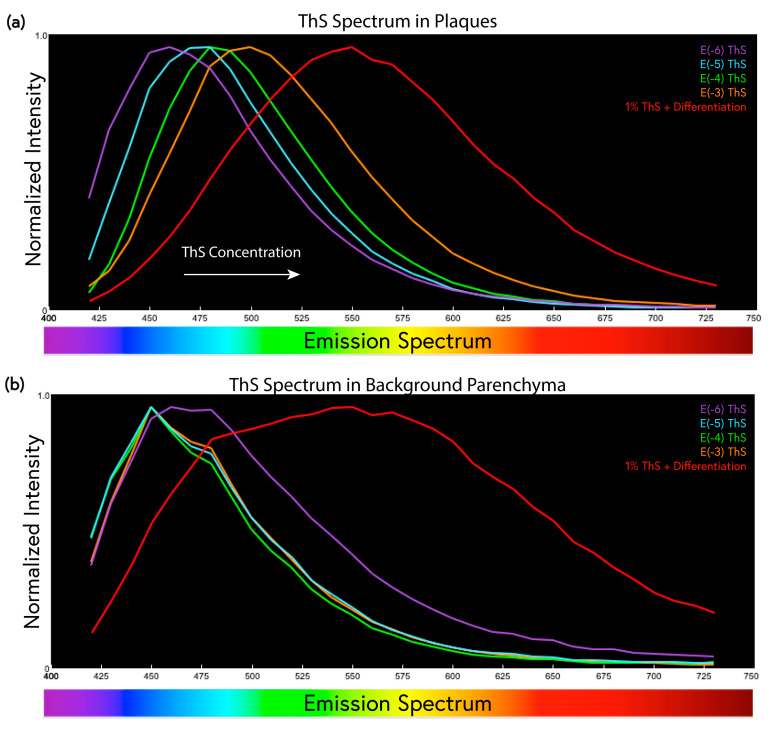
ThS emission spectra from plaques and 5xFAD background at various dye concentrations. (**a**) Normalized graph comparing the spectral shift of plaques stained with ThS from 1% to 1 × 10^−6^%. The emission spectrum red-shifted by ≈90 nm as the dye concentration was increased to 1%. (**b**) The non-plaque background spectrum remained quite blue at ≈450 nm at low ThS concentrations, except at 1 × 10^−6^% when most of the signal was tissue autofluorescence rather than ThS signal (see Figure 3a). The emission spectrum of the 1% ThS stained parenchyma had a broad and more variable spectrum than those of the plaques.

Not only did relative fluorescence intensities vary significantly depending on the concentration of dye used, but the fluorescence emission spectra of the ThS also exhibited marked differences. Normalized spectra at various ThS concentrations were plotted to better characterize the changes in the ThS spectra of the plaques and background parenchyma. Amyloid plaques became increasingly red-shifted as the ThS staining concentration was increased from 1 × 10^−6^ to 1% (Figure 4a); indeed, there was a surprisingly large 90 nm shift in the peak position between the lowest and highest concentration. The emission spectrum of the background parenchyma was similarly red-shifted, but only at the highest (1%) concentration; unlike plaques, the non-plaque background spectrum (representing both green/yellow parenchyma and redder cell bodies) remained constant at the lower concentrations (Figure 4b). At 1 × 10^−6^% ThS, the background parenchyma again began to red-shift as the ThS staining did not rise significantly above the level of intrinsic autofluorescence, which tends to emit in the 450–530 nm range (Figure 4b). 

### 2.3. ThS Fluorescence Intensity as a Function of Concentration

**Figure 5:**
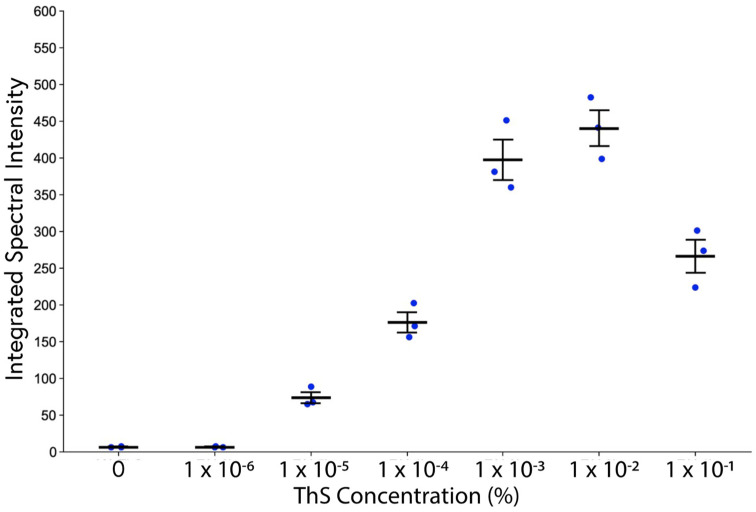
Integrated spectral intensity of plaque cores as a function of ThS concentration. Adjacent 5xFAD brain sections from the same mouse stained with various concentrations of ThS were all imaged with the same instrument settings. Plaque regions were selected in the images and the integrated spectral intensity was calculated. Maximum fluorescence intensity was achieved at 1 × 10^−2^% and unexpectedly diminished at the highest concentration tested. Error bars represent SEM.

We then examined the relationship between the absolute fluorescence intensity of the plaque cores and the concentration of ThS used for staining. Care was taken to ensure that instrument settings were kept constant for all samples at each concentration. As expected, the intensity of the plaques increased with ThS concentration. However, interestingly, this relationship was not monotonic; 1 × 10^−1^% unexpectedly resulted in markedly lower absolute plaque brightness compared with the maximum reached at 1 × 10^−2^% (Figure 5). 

### 2.4. Low-Concentration ThS Staining Detects Early Amyloid Pathology in Young 5xFAD Mice

**Figure 6:**
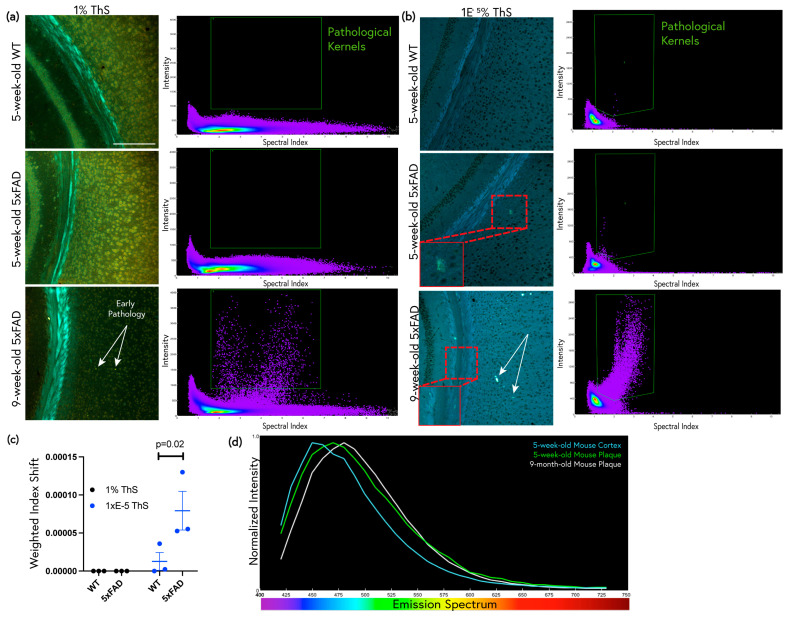
Optimized ThS staining detects early pathology in 5xFAD mice as young as 5 weeks of age. (**a**) 5-week-old WT and 5xFAD brain tissue sections and 9-week-old 5xFAD brain tissue sections labeled with 1% ThS for 10 min followed by ethanol differentiation. Pathology is neither visible nor apparent with spectral scatter analysis (SSA) at 5 weeks. Green boxes on SSA graphs indicate the regions where plaque kernels are located in the 9-month-old 5xFAD mouse and also indicate many plaque kernels that can be observed at 9 weeks. Scale bar: 200 μm. (**b**) Adjacent tissue sections from the same mice labeled with 1 × 10^−5^% ThS. Occasional sparse, dim amyloid-like deposits were seen in the cortex at 5 weeks, along with pathological kernels (green ROI) on SSA graphs. At 9 weeks of age, more prominent plaque-like deposits were apparent in the 5xFAD mouse brain when using both ThS staining protocols, but 1% ThS did not reveal early white matter threads (inset). (**c**) The kernels present in the green ROI on each SSA in (**a**,**b**) were plotted, and they show a significant shift in the weighted spectral index of the amyloid deposits compared with that of the WT that was only seen in sections stained with 1 × 10^−5^% ThS. Error bars represent SEM. (**d**) Normalized graph comparing spectral shift of 5-week-old 5xFAD mouse plaques and cortex to 9-week-old 5xFAD plaques. The spectrum of early forming pathology shifted towards that of fully formed plaques in the adult mouse.

Given that 1 × 10^−5^% ThS was found to be the most sensitive for subtle pathology in aged 5xFAD mice, this optimized protocol was then applied to 5-week-old 5xFAD and WT mice. Typically, 5xFAD mice begin to develop amyloid pathology at around 6 weeks of age [31]. In staining the tissues of even younger mice, we sought to test the sensitivity of ThS staining for very early, diffuse pathology by comparing 1% ThS + differentiation vs. 1 × 10^−5^% ThS staining per our protocol. The conventional 1% differentiation staining of the 5-week-old mouse brains did not reveal any pathological features visually (i.e., no obvious plaques were visible at this young age), and neither were any pathological features visible with spectral scatter analysis (Figure 6a). With 1% ThS, plaque pathology only became apparent once the 5xFAD mice were 9 weeks old. In contrast, 1 × 10^−5^% ThS revealed occasional cortical deposits with spectra very similar to those of amyloid plaques in the 5-week-old mice (Figure 6b,c). Upon examination of the brain tissues of the 9-week-old mice with 1 × 10^−5^% ThS, not only were plaques apparent in the cortex, but white matter threads were also visible in the corpus callosum. The emission spectra of the plaques were examined in both the 5-week-old and 9-week-old mice. The plaques red-shifted as the age of the mouse increased (Figure 6d). 

### 2.5. Low-Concentration ThS Staining Revealed Widespread Subtle Amyloid Deposition in the Non-Plaque Parenchyma

**Figure 7:**
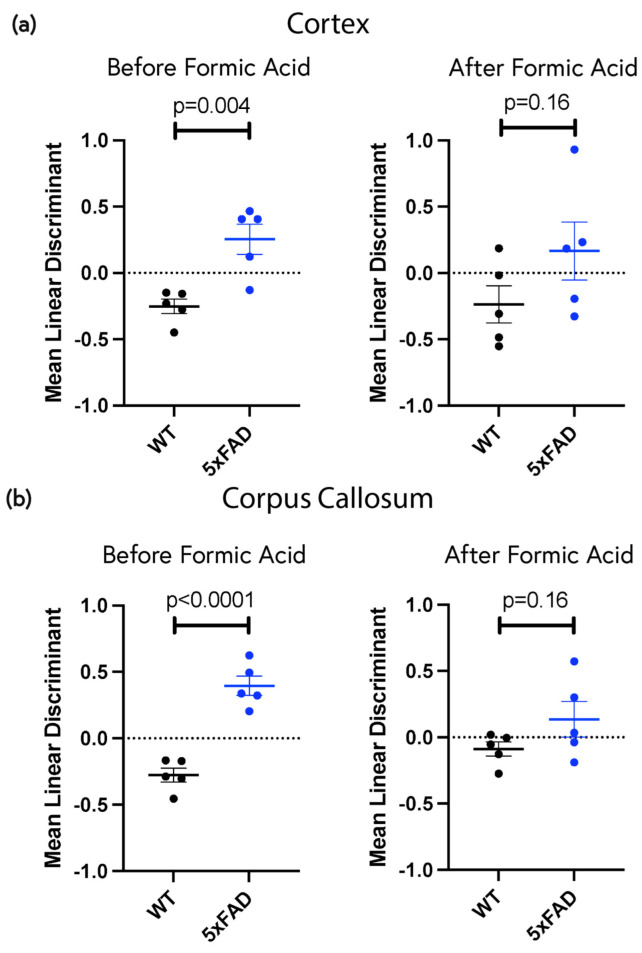
Linear discriminant analysis reveals significant spectral differences in background of 9-month-old WT vs. 5xFAD brain stained with 1 × 10^−5^% ThS that is sensitive to formic acid treatment. Linear discriminants were calculated from the spectrum of each image kernel, then averaged for each mouse and plotted. Higher linear discriminant values indicate a more pathological state. (**a**) Cortical background excluding all plaque regions. (**b**) Corpus callosum. Each dot represents a different mouse. *p* values calculated using Student’s *t*-test.

While plaque pathology could be readily visualized by eye, especially at lower ThS concentrations that yielded high plaque-to-background contrasts, we asked whether amyloid pathology was more widespread in the parenchyma, extending beyond plaque regions. Sections were stained with 1 × 10^−5^% ThS and images were analyzed by comparing spectra from the cortex or corpus callosum of 9-month-old WT and 5xFAD (all plaques excluded in the latter) mice using linear discriminant analysis. We found significant spectral differences between WT and 5xFAD in both the cortex and corpus callosum which were eliminated after formic acid treatment (Figure 7a,b). Taken together, these data strongly suggest a subtle, diffuse amyloid pathology in both the cortex and white matter of 5xFAD mice that is distinct from the more obvious conventional plaque deposits. 

**Figure 8:**
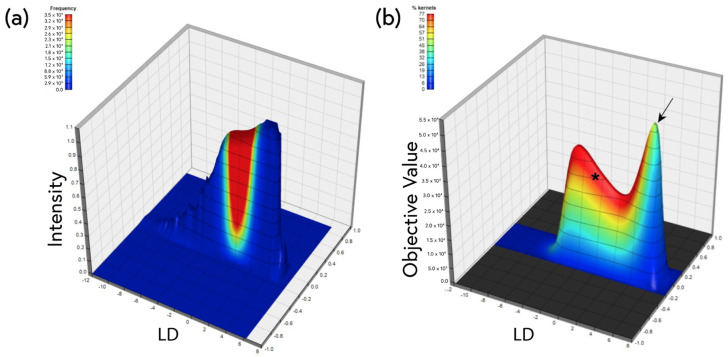
(**a**) Example surface plot representing a histogram of kernel-wise linear discriminants calculated from spectral micrographs of WT and 5xFAD mouse brains stained using 1 × 10^−5^% ThS. Regions of interest were scanned across 10 such surfaces (*n* = 5 for WT and *n* = 5 for 5xFAD) in an automated fashion, and inter-group statistics were calculated in an effort to identify smaller subpopulations of kernels (the pixels in the images) that exhibit even greater WT vs. 5xFAD differences. The result of such a surface scan is shown in (**b**). Two distinct peaks can be observed, the first (*) representing the bulk of the kernel population, and the second representing a smaller subpopulation of kernels (arrow) with a higher objective value (greater inter-group statistical difference). All plotted solutions had inter-group differences at the *p* < 0.05 level, but the peak solution showed a WT vs. 5xFAD difference at *p* = 0.0035 (Student’s *t*-test).

The analysis in Figure 7 included all background image kernels and therefore reflects the overall spectral shift of the background parenchyma. This assumes that changes are homogeneous, but it is possible that amyloid deposits, and therefore spectral shifts, could occur in discrete islands of exaggerated pathology surrounded by more normal regions. To explore this possibility, per-kernel linear discriminants were graphed on a surface plot and subregions of scanned image kernels and inter-group statistics were extracted (Figure 8a). Figure 8b shows a solution surface that reveals two distinct peaks in the cortex: a broader set (*) representing the majority of kernels, analogous to the all-kernels LDA in Figure 7, and a second narrower peak (arrow) indicative of a smaller percentage of the total kernels from the cortex, but with a higher “objective value” (a measure of statistical difference between the WT and the 5xFAD cortex). A similar bimodal solution was not observed in the corpus callosum (not shown), indicating a more homogeneous difference in that region and a lack of focal heterogeneities. Taken together, these results indicate that while the entire cortical background is spectrally different in WT vs. 5xFAD, there exist tiny subregions with a more exaggerated spectral shift that likely represent small foci of excess amyloid deposition, underscoring the heterogeneity even in a uniform-appearing background parenchyma. This was not the case in the corpus callosum. 

## 3. Discussion

This study explored the efficacy of a novel staining protocol using the well-known fluorescent probe ThS for the more sensitive detection of amyloid deposits in a mouse model of Alzheimer’s disease. ThS is commonly used to identify AD amyloid histology in addition to other amyloid fibrils, including tau tangles, α-synuclein, and insulin [34,35,36]. The fluorescent properties of ThS have also been utilized for purposes other than staining amyloid plaques, e.g., for the detection of deposits in the retina, for the detection of bacterial populations, and for the structural analysis of fibrils [21,37,38]. Historically, ThS staining involved applying relatively high concentrations of dye onto samples, incubating them for a short duration, then removing excess dye with differentiation washes [23]. While this technique has been widely used for histology in AD and other protein misfolding disorders for decades, high staining levels in non-plaque structures, variable differentiation, and non-specific tissue labeling may compromise the practitioner’s ability to reliably detect subtle amyloid deposition in a quantitative manner. Importantly, the capacity of ThS to shift its emission spectrum in response to its environment has not been well studied, and this could provide a very useful additional dimension that may enhance this dye’s performance as a probe for amyloid detection.

Traditional analysis of ThS staining solely involves measuring positivity by the intensity of the ThS signal. However, the use of its spectral properties to detect amyloid pathology has great potential. We combined fluorescence spectroscopy with ThS staining to explore its spectral properties upon binding to amyloid deposits in 5xFAD mouse brains, a widely used mouse model of AD. First, we characterized the features of the standard 1% differentiation staining using confocal microscopy and fluorescence spectroscopy (Figure 1). The ThS identified bright plaque cores as expected, but also resulted in the substantial staining of white matter, even in age-matched WT mice. Spectral imaging revealed further visual differences between the white matter and the plaques, in addition to red-shifted cell bodies that were stained throughout the greater parenchyma (Figure 2a). A spectral scatter analysis of these staining features enabled the separation of the spectra from these structures, isolating them based on both intensity and spectral shift (Figure 2b). Using these methods, richer, more informative data could be collected, and the issues caused by high levels of non-plaque staining were mitigated by resolving features along the spectral dimension. It is possible that meaningful spectral differences between WT and 5xFAD white matter or non-plaque grey matter existed. However, the short incubation time and ethanol differentiation may have resulted in spectral discrepancies from sample to sample (and within the same tissue sample) that were difficult to control.

Next, we optimized the ThS protocol with a focus on improving both its staining consistency and its sensitivity to subtle, diffuse pathology. By using controlled concentrations of ThS well below the traditional levels together with spectral detection, the non-specific background and white matter ThS signal was reduced, and amyloid pathology could be more readily identified via both intensity vs. background signal and shifted fluorescence emission spectrum. This enabled the identification of far more amyloid pathology than the commonly employed 1% ThS/differentiation method (Figure 3b,c), suggesting that standard ThS staining protocols may vastly underestimate the severity of amyloid deposition in AD, and possibly in other neurodegenerative diseases. Furthermore, we observed a decrease in the fluorescence intensity of plaques with high ThS levels (Figure 5). This nonmonotonic relationship between ThS binding and its emission intensity may be a result of self-quenching behaviour at high concentrations that could lead to a loss of sensitivity even to the more obvious plaque pathologies, as has been observed with ThT [39]. 

The emission spectrum of the dye in the plaques and background parenchyma also changed as a function of concentration. From the lowest concentration tested (1 × 10^−6^%) to the traditional 1% staining, there was a substantial 90 nm shift in the peak emission spectrum of the plaques (Figure 4a). This could be due to the heterogeneity of the compounds present in the ThS, which may contribute more to the fluorescence emission at higher concentrations, or due to molecule–molecule interactions within the amyloid plaque pockets, e.g., the formation of excimers at higher ThS staining concentrations. These phenomena should be taken into careful consideration when optimizing ThS and other probes for the optimal detection of amyloids in tissue samples. 

Staining the tissues in Coplin jars at low concentrations overnight resulted in a controlled method for creating even and reproducible levels of ThS staining and did not require subjective differentiation. Other amyloid probes, such as ThT or the array of available oligothiophenes, could be similarly titrated for optimal sensitivity. Surprisingly, the optimized ThS staining procedure detected amyloid pathology in 5xFAD mice as young as 5 weeks of age (far earlier than the standard 1% ThS protocol), further underscoring the high sensitivity and specificity of our improved method. Furthermore, the ThS revealed widespread white matter threads in the corpora callosa of 5xFAD mice as young as 9 weeks of age that were not visible using the standard protocol (see Figure 5). Future studies could attempt to further optimize the protocol by exploring shorter staining times. The sensitive detection of amyloid pathology in the white matter is relevant because neuroimaging studies of preclinical AD demonstrate widespread white matter abnormalities despite unremarkable grey matter [40].

Lastly, we examined the non-plaque background regions of the cortex to determine whether diffuse widespread spectral changes were also present in the greater parenchyma of the aged 5xFAD mouse. Spectral patterns in images are complex and often subtle, making it difficult to distinguish healthy tissue from diseased tissue either by eye or using conventional methods. Machine learning was thus employed to examine the emission spectra on a pixel-by-pixel basis. LDA extracted the quantitative spectral features from the 5xFAD and WT images and identified significant differences in spectral emission patterns between the two groups (Figure 7). In order to further support the notion that the detected differences were due to amyloid deposition, we treated the samples with formic acid, which is known to disrupt β-amyloid motifs and thus disassemble amyloid aggregates and reduce specific probe binding [41]. An analysis of the formic acid-treated samples revealed that the inter-group differences in spectral patterns were abolished, supporting the idea that the ThS had detected amyloid deposition in the samples not exposed to formic acid. The ability to detect these subtle yet widespread changes in the brain are potentially relevant to human AD as the soluble pool of β-amyloids is more strongly associated with disease severity than the insoluble aggregates that can be detected using conventional histological methods [42]. The ability to detect subtle amyloid deposition in the earliest stages of AD would potentially allow early interventions to lower rates of β-amyloid accumulation in the brain and thus reduce disease burden.

## 4. Materials and Methods

### 4.1. Animals and Animal Care 

All animal experiments were approved by the Animal Care Committee at the University of Calgary using standards set out by the Canadian Council on Animal Care. A 5xFAD mouse colony (Tg6799, stock number 34,840-JAX, Jackson Laboratory, Bar Harbor, ME, USA) was maintained by crossing heterozygous transgenic mice with wild type (WT) mice, and genotyping performed using PCR analyses of ear notch samples.

### 4.2. Mouse Tissue Processing

Nine-month-old male and female 5xFAD and WT mice were deeply anesthetized using sodium pentobarbital and transcardially perfused using PBS followed by 4% paraformaldehyde. The harvested brains were fixed in 4% paraformaldehyde for 24 h. The tissues were subsequently transferred to 20% sucrose overnight, then to 30% sucrose overnight. The tissues were then frozen and cryosectioned onto VWR Superfrost Plus slides using a cryostat at 20 μm thickness (3 coronal brain sections per slide). Sectioned tissues were stored at −20 °C until further analysis. 

### 4.3. Staining Procedure

ThS was obtained from MP Biomedicals, LLC (cat. number 218766), and filtered 1% stock solutions were prepared in distilled water and stored at −20 °C. Since the chemical composition of ThS is poorly defined, ThS concentrations were reported according to their weight/volume percentages rather than their molarity. The mouse brain sections were first rinsed using PBS to remove any residual mounting medium. The initial staining protocol involved incubating slides in 1% ThS for 10 min at room temperature and protected from light, followed by a series of ethanol differentiation steps (80%, 80%, and 95% ethanol for 3 min each) and 3 washes in distilled water before coverslipping [23,43]. To optimize the staining protocol, tissues were instead incubated in a range of ThS concentrations (1 × 10^−6^% to 1 × 10^−2^% [g/100 mL]) in PBS in 50 mL glass staining jars on a shaker at 55 RPM for 24 h at room temperature and protected from light. The sections were then rinsed in PBS and mounted in 50% PBS:glycerol. After spectral confocal imaging of the 5xFAD and WT tissues, the coverslips were removed and the samples were incubated with 90% formic acid for 30 min to disrupt β-sheet structures, rinsed in PBS, re-stained for 24 h in a new solution of ThS, and then re-mounted in 50% PBS:glycerol and imaged again.

When the same glass staining jars were reused for several experiments, the tendency of residual ThS to remain in the staining jars despite vigorous washing became apparent. To combat this problem, especially when using very low concentrations of ThS for tissue staining, a unique jar was assigned to each concentration, and the jars were incubated overnight in 100% ethanol after each use, followed by rinsing with bleach and soapy water.

### 4.4. Imaging and Analysis

Spectral images were acquired on an inverted C1si confocal laser scanning microscope (Nikon) using a 20× water objective with a numerical aperture of 0.75 and an excitation wavelength of 404 nm. The emission spectra were collected between 415 nm and 735 nm in 10 nm increments using a Nikon C1si spectral detector with a multi-anode photomultiplier array. Three images of the cortical grey matter were taken per mouse. The collected files were converted to 32-channel spectral images and analyzed using ImageTrak software, version 5.2.0b (available for download at https://stysneurolab.org/imagetrak). To quantify amyloid pathology by spectrum, the emission spectrum of each kernel (2 × 2 pixels) in each image was analyzed using spectral scatter analysis, wherein kernels were plotted as a function of intensity and mean spectral index (a low index representing a bluer emission and a high index representing a redder emission). High-intensity kernel populations were isolated, and the associated pixels were then masked on each image. The number of identified pixels in the blue masked area compared with the whole image was plotted to compare the amount of amyloid pathology identified at each ThS concentration.

The absolute intensity of the amyloid plaque cores was determined by first imaging tissue sections stained along a ThS concentration curve (1 × 10^−6^% to 1 × 10^−1^%). All images were taken at the same laser power, pixel dwell time, pinhole size, and detector gain to allow for a direct comparison of intensity. The image pixels were averaged into 2 × 2 kernels to improve signal-to-noise and then plotted on spectral scatter analysis graphs where the *x*-axis represented spectral shape (higher index values indicate a more red-shifted spectrum) and the *y*-axis represented intensity. Blue pixel masks were applied to images of selected features of interest, and the integrated spectral intensity was taken for each image.

### 4.5. Machine Learning

The WT and 5xFAD data sets were analyzed using linear discriminant analysis (LDA). LDA determines a linear combination of features to separate two or more classes of subjects [44], in this case WT and 5xFAD. The resulting combination was used to reduce the dimensionality of the spectral data for the classification of the images [45]. A linear discriminant was calculated for each kernel in the image using scikit-learn’s LinearDiscriminantAnalysis package [46]. Each mouse was assigned a class and plotted as an average of all linear discriminants for each kernel in an image, with three images acquired per mouse.

## 5. Conclusions

By modifying the long-standing protocol for ThS and using spectral detection, the performance of this dye in identifying diffuse amyloid pathology in the 5xFAD mouse model of Alzheimer’s disease was significantly improved. This approach could also be used to improve the detection of amyloid pathology in other proteopathies, or to optimize the sensitivity and specificity of other amyloid probes. 

## Data Availability

Original data can be provided upon reasonable request.
